# Integrated Care and Population Health Management; Two Sides of the Same Coin?

**DOI:** 10.5334/ijic.8922

**Published:** 2024-06-13

**Authors:** Annefrans F. T. M. van Ede, K. Viktoria Stein, Marc A. Bruijnzeels

**Affiliations:** 1 Health Campus The Hague/Department of Public Health and Primary Care, Leiden University Medical Centre, The Hague, The Netherlands

**Keywords:** Population Health Management, Integrated Care, Practice, Theory

The predecessor of IFIC (International Foundation for Integrated Care) was founded in 2000, and since that date the network advocating for Integrated Care (IC) has grown to a global movement for change. In recent years, the concept of Population Health Management (PHM) has come up using similar terminology and aspiring well-known outcomes. As both concepts continue to gain traction with policy makers and practitioners, the question of similarity and difference has arisen in research and practice. Are IC and PHM just synonyms for the same underlying approach or are they fundamentally different?

This year, the International Conference on Integrated Care was held in April in Belfast Northern Ireland. Accompanied by some sun and clouds, many international scholars, health professionals, and people with lived experience shared their experience and expertise on Integrated Care and discussed new ways of moving forward. One of these lively discussions took place during the workshop on the difference between IC and PHM. Not being held back by the early morning start on the last day of the conference quite a large audience of 50 people joined the discussion between the two main discussants, Viktoria Stein (IC) and Marc Bruijnzeels (PHM), who each advocated for their own term regarding 4 statements: PHM is data driven, IC is not; IC focusses on service integration, PHM does not; PHM is health centered, IC is disease centered; and IC is for care professionals, PHM is for managers/executives. The audience had to vote with their feet by positioning themselves along an imaginary scale in the room.

What became increasingly clear throughout the workshop is the difference between theory and practice. As one of the participants articulated: “Both IC and PHM are concepts, frameworks, models; it is an uncomplete attempt of the human mind to understand complex reality.” This might be one of the hardest challenges we all face within the implementation of change. How to get brilliant ideas to actually work out in reality? Comparing IC to PHM, it is important to stress if you are comparing ideal conceptual processes encompassed in the term or how it worked out thus far in practice. From a conceptual perspective, differences between both could be exaggerated, which has meaning in trying to understand the complex reality.

Also, what arose during the workshop was the journey that both terms had experienced over time, as they both changed scale and scope considerably with each iteration of implementation (and failure). IC started out as disease management for the people with the highest care needs but is now used more broadly to describe the integration of the health and social care sectors and beyond. Due to the stronger focus on social needs and the availability of data capabilities, IC embraces these aspects in addition to the ambition to organise continuous processes of care around individuals and populations. The popularity of PHM is more recent and started out with a strong focus on whole populations and the segmentation and risk stratification within populations, very quantitatively data-driven, but now PHM covers more areas including prerequisites for implementation and qualitative data [1].

Besides the conceptual differences, in practice some differences were noted as well. IC started in the early days often with attempts of professionals to organize care more smoothly, whether in combination with social care or within highly specialized facilities. Through the learnings from these service integration initiatives, the need for expanding the focus to other areas of integration (professional, organizational and system) was noted [2]. PHM gained its popularity through the focus on the system level: due to the organizational fragmentation and lack of focus on populations many improvements failed [3]. What was discussed in the workshop was who do you need for change? Who do you need to focus on? The general consensus was that first and foremost, IC is about bringing the professionals together, bringing teams together across sectors, across professions; and, creating a level playing field to collaborate with the community. However, we also need the managerial and the executive people to be on board. We need them to form a team, an alliance or a network, and we need the data competences of the different organizations to be brought together so that the care professionals can keep doing what they are good at. One participant framed it thus: “Something is always easier if you have a clear vision and if everybody has bought into that vision.” So, you need your care professionals and your managers/executives to be all working together on the same side of the coin. But how do you convince these managers?

All participants agreed that what PHM brings is the focus on data, which can be a convincing argument. This should not only be analyses of quantitative data, but also the narratives of the people, patients, and populations. A solid mixed methods methodology is needed to apply PHM well [4]. Successful initiatives in practice went into the community with a collective co-design process before actually designing the program, followed by an analysis of quantitative data using segmentation, risk stratification, and impactability modelling. Only then should you start creating and implementing tailored services to improve the health of the population. Furthermore, both quantitative and qualitative data should be used continuously for evaluation purposes. This data focus is emphasized by PHM and brought into the IC arena. Very important in this regard is to not lose sight on who it is you are doing it with.

What did we learn from this workshop? PHM and IC look much more similar in practice than in theory. As one of the participants wondered: “Is it the right question to be asking if they are different or not?” For many people it does not matter what it exactly is called what we are trying to do as long as we are on the same page on what it is we are doing with all people we are doing it with. The importance of creating a common language, of being aware of the different meanings of technical terms and the detrimental impact a lack of clarity can have on the implementation process was emphasized by many participants. For academics, a more nuanced exploration of the use of PHM and IC and their conceptual differences seems needed for the understanding of mechanisms and outcomes of the factual principles of both. Particularly when building learning health systems by comparing and contrasting different initiatives, whether in practice or on paper, it is necessary to clarify what has happened and what strategy was used. Using terms consistently would support this.

So, as a conclusion to the workshop we can say that, in practice IC and PHM complement each other, whereas in science differences contribute to the understanding of the various mechanisms. The workshop highlighted the key challenges, which still remain in the implementation of change of any kind in the health and care systems, and the passion with which our audience advocate and work towards better outcomes. As any good scientific debate, we ended with the realization that we need to continue this discussion as many questions remain unanswered, but one message was clear: in order to convince people to change you need to be clear about your vision and you need to win their hearts and heads. This discussion also leaves questions open to the IC research community and to the journal. If IC and PHM indeed are different, should they be applied both, under what circumstances, in which contexts? Should the journal encourage more theoretical articles on the differences and similarities of both concepts? So, up to the next IFIC conference to continue our journey on IC and PHM.
